# The Dual Role of Nrf2 in Nonalcoholic Fatty Liver Disease: Regulation of Antioxidant Defenses and Hepatic Lipid Metabolism

**DOI:** 10.1155/2015/597134

**Published:** 2015-05-18

**Authors:** Sílvia S. Chambel, Andreia Santos-Gonçalves, Tiago L. Duarte

**Affiliations:** ^1^Basic and Clinical Research on Iron Biology Group, Instituto de Biologia Molecular e Celular (IBMC), Universidade do Porto, Rua do Campo Alegre 823, 4150180 Porto, Portugal; ^2^Instituto de Investigação e Inovação em Saúde, Universidade do Porto, Portugal

## Abstract

Nonalcoholic fatty liver disease (NAFLD) is a progressive liver disease with ever-growing incidence in the industrialized world. It starts with the simple accumulation of lipids in the hepatocyte and can progress to the more severe nonalcoholic steatohepatitis (NASH), which is associated with inflammation, fibrosis, and cirrhosis. There is increasing awareness that reactive oxygen species and electrophiles are implicated in the pathogenesis of NASH. Transcription factor nuclear factor erythroid 2-related factor 2 (Nrf2) is a positive regulator of the expression of a battery of genes involved in the protection against oxidative/electrophilic stress. In rodents, Nrf2 is also known to participate in hepatic fatty acid metabolism, as a negative regulator of genes that promote hepatosteatosis. We review relevant evidence in the literature that these two mechanisms may contribute to the protective role of Nrf2 in the development of hepatic steatosis and in the progression to steatohepatitis, particularly in young animals. We propose that age may be a key to explain contradictory findings in the literature. In summary, Nrf2 mediates the crosstalk between lipid metabolism and antioxidant defense mechanisms in experimental models of NAFLD, and the nutritional or pharmacological induction of Nrf2 represents a promising potential new strategy for its prevention and treatment.

## 1. Hepatic Lipid Metabolism

The liver plays a key role in the processing of lipids, including the synthesis and degradation/oxidation of fatty acids (FA), and the metabolism of cholesterol and phospholipids. Hepatocytes convert the excess dietary glucose into FAs (lipogenesis), which can be stored as triglycerides (TGs) in lipid droplets or used in the generation of phospholipids [1, 2]. Lipogenesis involves acetyl-CoA precursors and is an insulin-dependent process. Under normal conditions, TGs along with cholesterol and phospholipids are assembled into very low density lipoprotein (VLDL) particles that can be secreted into the bloodstream for storage in other tissues in the form of lipid droplets, thus preventing TG accumulation in the cytoplasm of hepatocytes [1, 3]. On the other hand, when the available glucose cannot meet the energy demands, hepatocytes break down TGs and cholesterol stored in lipid droplets through a lysosomal degradative pathway of macroautophagy designated lipophagy. The breakdown of TGs by lipophagy supplies free fatty acids (FFAs) required to sustain rates of mitochondrial *β*-oxidation for the generation of ATP [4]. FA degradation occurs also in peroxisomes (*β*-oxidation) and in the endoplasmatic reticulum (ER) (*ω*-oxidation).

When fatty acid input exceeds the capacity of *β*-oxidation, accumulating acyl-CoA is drained by triglyceride synthesis, leading to the supraphysiological accumulation of fat in hepatocytes (hepatosteatosis), which is a hallmark of both alcoholic and nonalcoholic fatty liver disease (NAFLD) [5]. Hepatic steatosis increases the oxidation of FFAs and the rate of the tricarboxylic acid cycle. Increased FFA *β*-oxidation results in increased rates of electron leakage from the mitochondrial respiratory chain [6], resulting in higher free radical formation, and increases hydrogen peroxide production in the peroxisomes. A decrease in the mitochondrial quinone pool and the associated reduction of mitochondrial oxidation metabolism were also proposed to account for the increased mitochondrial reactive oxygen species (ROS) production under high-fat diet (HFD) [7]. Microsomal oxidation also contributes to oxidative stress via the activity of cytochromes P450 2E1 and P450 4A. The incorporation of free saturated fatty acids, the first products of* de novo* lipogenesis, into membrane phospholipids is detrimental to the ER and leads to Ca^2+^ release, which has the potential to injure adjacent mitochondria and promote apoptotic cell death (lipoapoptosis) [2, 8].

## 2. NAFLD: Prevalence and Etiology

The increase in obesity has become an alarming public health trend in the industrialized world, with a growing burden on health care costs [9, 10]. Obesity leads to an increased propensity for the development of a complex array of factors that increase the risk to develop cardiovascular disease or type 2 diabetes, which have been termed the metabolic syndrome (abdominal obesity, atherogenic dyslipidemia, hypertension, and insulin resistance) [11]. NAFLD is the hepatic manifestation of the metabolic syndrome. In western countries, NAFLD is estimated to affect 20–30% of the general population, although the incidence may be even higher in obese individuals. The disease is highly correlated to obesity. It starts with the relatively benign accumulation of lipids in the hepatocyte. In up to one-third of patients, steatosis can progress to the more severe nonalcoholic steatohepatitis (NASH), which is associated with inflammation, fibrosis, and impaired liver function (cirrhosis) [12].

The detailed pathological mechanisms leading to the transition from lipid deposition to necroinflammation and cytotoxicity remain unclear, but a “two-hit” theory is widely advocated in the literature [14]. The first hit corresponds to “simple” hepatocellular lipid accumulation (steatosis) resulting from increased inflow of FFAs derived from insulin resistant adipose tissue, increased hepatic* de novo* lipogenesis, or impaired lipid export from hepatocytes. The second hit may be a consequence of increased ROS production in the liver resulting from the increased metabolism of FAs, resulting in mitochondrial dysfunction, upregulation of proinflammatory cytokines, and activation of hepatic stellate cells, which begin to proliferate and increase production of collagen, causing fibrosis. There is nevertheless growing evidence that the development of NAFLD and the progression to NASH are more complex processes than initially predicted by the two-hit theory. For instance, while hepatic TG formation is an early indicator of liver metabolic stress and disease, it is unlikely to be the initiating step in NASH. Instead, TGs may represent harmless storage compartments that divert FFAs away from potentially toxic pathways (e.g., microsomal oxidation), thus protecting hepatocytes from lipoapoptosis [5, 8, 15]. Moreover, multiple mechanisms/factors may contribute to the progression of steatosis to NASH (second hit), including increased ROS production in the liver (via activation of inflammatory cells, mitochondrial dysfunction, or the uncoupling of cytochrome P450 2E1 and 4A isoenzymes), the failure of hepatocytes to synthesize sufficient endogenous antioxidants, ER stress, liver and adipose tissue inflammation, decreased autophagic function, and/or the mild hepatic iron overload that is frequently seen in NAFLD patients [8, 16, 17].

## 3. Nrf2 Activation by Hepatic Oxidants/Electrophiles

The liver has relatively high metabolic activity and is the main organ responsible for the biotransformation and subsequent detoxification of xenobiotics. These properties place the organ at an increased risk for exposure to ROS [2] and electrophiles [30] which, in turn, are increasingly implicated in the pathogenesis of NAFLD and other chronic liver diseases. In the hepatocyte, the major sites of ROS production are the mitochondria and the cytochrome P450 system. Electrophiles are produced by oxidation and nitration of unsaturated FAs, resulting in a series of reactive species, including *α*,*β*-unsaturated aldehydes such as 4-hydroxynonenal (4-HNE) [30]. Hepatocytes are equipped with multiple defense systems that ensure protection against the toxic effects of endogenous and exogenous oxidants and electrophiles to which they are exposed, including (a) phase 1 enzymes that introduce functional groups onto largely hydrophobic organic molecules, such as cytochrome P450 enzymes, whose activity may produce highly reactive products that are toxic to the cell; (b) phase 2 enzymes like glutathione S-transferases and UDP glucuronosyl transferases, which conjugate the products of phase 1 enzymes with hydrophilic groups in order to facilitate their excretion, and antioxidant enzymes like superoxide dismutases, glutathione peroxidase, and catalase, which inactivate ROS; (c) phase 3 efflux transporters that export toxic metabolites acting synergistically with phase 2 enzymes to provide protection against electrophiles and carcinogens; and (d) thiol containing molecules such as glutathione and thioredoxin that function to maintain reducing conditions within the cell and inactivate electrophilic compounds [31]. Importantly, many of these cytoprotective enzymes are encoded by genes containing antioxidant response elements (AREs) in their promoter regions. AREs were initially identified as 5′(G/A)TGA(G/C)nnnCG(G/A)3′ [32] and subsequently expanded to 5′TMAnnRTGAYnnnGCRwwww3′, where M = A or C, R = A or G, Y = C or T, W = A or T, and S = G or C [33].

**Figure 1:**
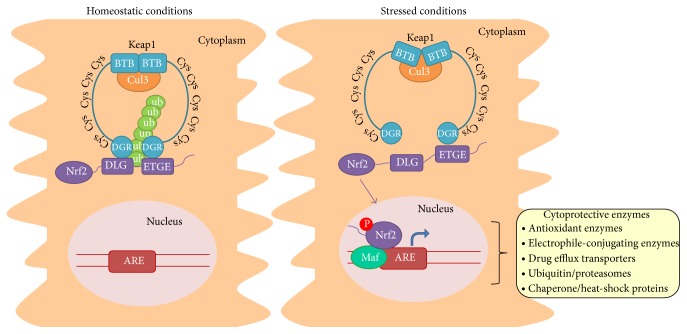
Activation of the Keap1-Nrf2-ARE pathway by oxidants/electrophiles. Under homeostatic conditions, Nrf2 is mainly localized in the cytoplasm through an interaction with Keap1 and the actin cytoskeleton. Keap1 is a five-domain protein consisting of an N-terminal broad complex, Tramtrack and Bric-à-brac (BTB) domain, an intervening region with cysteine (Cys) residues, a C-terminal Kelch domain with double glycine repeats (DGR), and the C-terminal domain. Keap1 homodimerizes at the BTB domain, which is also a binding site for Cullin 3 (Cul3). The Keap1 homodimer binds to a single Nrf2 molecule through the ETGE and DLG motifs of Nrf2, each binding to a DGR domain in Keap1. According to the proposed hinge and latch model [13], ETGE is a high-affinity motif (“hinge”) whereas DLG is a low-affinity one (“latch”). Keap1 functions as an adaptor protein in the Cul3-based E3 ligase complex, which results in the polyubiquitination (Ub) of the lysine residues situated between the DLG and ETGE motifs, and subsequent proteasomic degradation of Nrf2. Under stressed conditions, the modification of critical cysteine residues of Keap1 destabilizes its binding to the DLG motif of Nrf2, which blocks ubiquitination/proteasomal degradation and allows Nrf2 to escape Keap1 control and translocate into the nucleus. In the nucleus, Nrf2 heterodimerizes with small Maf proteins and promotes the expression of ARE-containing genes involved in cell stress response, drug metabolism, detoxification, and transport. Nrf2 may also be phosphorylated (P) by stress-activated kinases.

The ARE is a* cis*-acting enhancer sequence that mediates transcriptional activation of genes in response to changes in the cellular redox status, such as during increased production of free radical species, or to prooxidant xenobiotics that are thiol reactive and mimic an oxidative insult [34]. Transcription factor nuclear factor-erythroid 2-related factor 2 (Nfe2l2/Nrf2) is a basic leucine zipper transcription factor that regulates transcriptional induction of ARE-containing genes encoding antioxidant enzymes, electrophile-conjugating enzymes, ubiquitin/proteasomes, and chaperone and heat-shock proteins in response to cellular stresses including ROS [35, 36]. Under normal conditions, Nrf2 is mainly localized in the cytoplasm through an interaction with Kelch ECH associating protein 1 (Keap1) and the actin cytoskeleton. Despite the fact that Nrf2 mRNA is constitutively expressed, Keap1 targets Nrf2 for polyubiquitination and degradation, resulting in a short protein half-life. The binding to and regulation of Nrf2 by Keap1 have been explained by a “hinge and latch model” [13], as described in Figure 1. During exposure to electrophiles or oxidative stress, Keap1 becomes oxidized at critical cysteine residues. As a result, Nrf2 escapes Keap1 control and translocates to the nucleus, where it dimerizes with small musculoaponeurotic fibrosarcoma (Maf) proteins and promotes the expression of ARE-containing genes [36–38]. In recent years, it has become apparent that Nrf2 activity is not controlled exclusively through Keap1-mediated proteasomal degradation. In addition to Keap1, Nrf2 protein stability is regulated by another E3 ubiquitin ligase adaptor, *β*-transducin repeat-containing protein (*β*-TrCP). Nrf2 degradation independent of Keap1 is promoted by glycogen synthase kinase 3 (GSK-3), which phosphorylates specific serine residues in the Neh6 domain of Nrf2 corresponding to the *β*-TrCP recognition motif [39]. Alternatively, Nrf2 activation may occur as a result of Nrf2 phosphorylation by mitogen activated protein kinase cascade, phosphatidylinositol 3-kinase, protein kinase C, and protein kinase RNA-like endoplasmic reticulum kinase (Perk) [36, 40].

In addition to the modulation of protein stability, Nrf2 activity is also regulated at the transcriptional level. Polycyclic aromatic hydrocarbons activate Nrf2 transcription through binding of the heterodimer formed by the aryl hydrocarbon receptor (AhR) and the AhR nuclear translocator to xenobiotic response element-like sequences in the Nrf2 promoter [41]. In certain tumor cells, activation of Nrf2 transcription by Kras oncogene is responsible for increased chemoresistance [42]. Moreover, basal Nrf2 activity seems to be regulated by epigenetic mechanisms and miRNA species, as reviewed elsewhere [43].

## 4. Nrf2 Protection against Liver Injury

As discussed, the Keap1-Nrf2-ARE pathway is activated in response to oxidative/electrophilic stress and regulates the basal and inducible expression levels of a battery of proteins involved in the detoxification of reactive molecules in the cytosol, mitochondria, and ER [30]. Overall, this transcriptional response protects cells against a series of insults and favors cell adaptation/survival [40, 44]. Cellular survival is also mediated by the UPR, which restores ER homeostasis and by the autophagy-lysosomal pathway, which promotes the degradation of proteins and dysfunctional organelles. There is increasing evidence that hepatic steatosis and ER stress are interconnected. This is not surprising since several enzymatic lipogenic pathways are located in the ER, including fatty acid elongation, cholesterol biosynthesis, complex lipid biosynthesis, and assembly of VLDL particles. In fact, abrogation of the UPR results in ER stress-induced development of steatosis [45]. During the UPR, Perk-dependent phosphorylation may also lead to Nrf2 nuclear translocation and increased transcription of Nrf2 target genes [40]. Nrf2 is also activated during autophagy, via interaction of the selective autophagy substrate p62 with the Nrf2 binding site on Keap1 [46]. Accumulation of p62 (as a consequence of a deficiency in autophagy) results in stabilization of Nrf2 and transcriptional activation of Nrf2 target genes [46]. A study by Kwon et al. demonstrated that high steady-state expression of NAD(P)H dehydrogenase, quinone 1, sustained by p62-induced basal Nrf2 activation, is required to maintain mitochondrial integrity [47]. Other studies have reported that Nrf2 deficiency results in mitochondrial depolarization, reduced ATP production, and decreased rate of oxygen consumption (mitochondrial respiration) [48], as well as less efficient mitochondrial fatty acid oxidation [49].

Several studies have demonstrated that Nrf2^−/−^ mice are more susceptible to chemical-induced oxidative/electrophilic stress in the liver than wild-type mice [50–52]. Nrf2 protects mice from 2,3,7,8-tetrachlorodibenzo-*p*-dioxin- (TCDD-) induced oxidative damage and steatohepatitis [53] and from hepatic fibrosis caused by chronic treatment with the hepatotoxin carbon tetrachloride [54]. Nrf2-deficient hepatocytes are also more susceptible to the toxicity of excessive iron accumulation [55].

## 5. Nrf2 in Liver Regeneration and Aging

The liver is the only organ in the human body capable of completely regenerating itself after injury. In the regenerating liver, hepatocytes accumulate a significant amount of lipids within lipid droplets, which are mainly composed of triacylglycerol and cholesteryl esters [56–58]. There is growing evidence that Nrf2 is required for effective tissue repair. Cell regeneration is diminished in hepatectomized Nrf2-null mice, which is associated with increased oxidative stress, reduced insulin/insulin growth factor-1 signaling [59], and reduced expression of the gene encoding a hepatotropic factor, augmenter of liver regeneration [60]. Importantly, the ability of the liver to regenerate after hepatectomy or chemical injury declines with old age [61]. Aging is also associated with increased lipid accumulation in the liver, which may ultimately result in lipotoxicity. Aging has been reported to increase the prevalence of the metabolic syndrome and of NAFLD in the human population and to enhance the progression to NASH and fibrosis [61]. Nrf2 plays an important role in the hepatic aging process. With age, there is a substantial reduction in glutathione (GSH) levels and in the expression and activity of glutamate cysteine ligase, the rate-controlling enzyme in GSH synthesis. This is accompanied by lower levels of Nrf2 protein and a reduction in Nrf2/ARE binding [62], as well as increased markers of protein and lipid oxidation [63]. Conversely, the liver of aged Nrf2-null mice shows lower free radical reducing activity [64] and GSH synthesis. The reason why aging organisms gradually lose the ability to activate Nrf2 is currently not understood [43], but a decline in Nrf2 signaling is presumed to contribute to the age-related hepatic oxidative stress. Whether it also contributes to the increased progression from NAFLD to NASH and fibrosis in the elderly is a subject that warrants further investigation.

## 6. Nrf2 Regulation of Hepatic Lipid Metabolism

Besides activating antioxidant and detoxification genes in response to electrophilic or oxidative stress, there is increasing evidence that Nrf2 participates in hepatic fatty acid metabolism (Table 1). A microarray study by Yates et al. [19] showed that the genetic or pharmacological activation of Nrf2 represses the expression of key enzymes involved in fatty acid synthesis, with concomitant reduction in the levels of hepatic lipids. A global analysis of constitutive hepatic protein expression in Nrf2-null and wild-type mice subsequently identified two main groups of Nrf2-regulated proteins. One group comprised proteins involved in phase II drug metabolism and antioxidant defense, for which expression was enhanced in the Nrf2 wild-type animals. The other group corresponded to proteins involved in the synthesis and metabolism of fatty acids and other lipids, and unlike proteins involved in the cellular defense, these proteins were expressed to a higher level in the Nrf2^−/−^ animals [20]. It is worth noting that both studies were performed with young adult mice (9-10 weeks of age). Another study performed with mice at 8 weeks of age has reported that, under control diet, mRNAs of sterol regulatory element-binding protein-1c and fatty acid synthase were more expressed in Nrf2-null animals than in the wild-type [18]. However, studies employing older mice (12–25 weeks of age) of the same genetic background (C57BL/6) showed that Nrf2 has little effect in hepatic fatty acid metabolism in animals fed control diets [21–23]. Studies in which mice received HFD have also reported that hepatic lipogenesis is negatively regulated by Nrf2 [21, 24]. Once again, studies using older mice (at approximately 6 months of age) either failed to detect an effect [23] or identified Nrf2 as an activator of genes involved in lipid synthesis and uptake (e.g., sterol regulatory element-binding protein, fatty acid synthase, stearoyl-CoA desaturase-1, and peroxisome proliferator-activated receptor-*γ*) via the induction of nuclear receptor small heterodimer partner (Shp/Nrob2) gene transcription and a downmodulator of genes regulating fatty acid oxidation (e.g., peroxisome proliferator-activated receptor-*α*, acetyl-CoA oxidase, and carnitine palmitoyltransferase 1*α*) [25]. It is worth noting that while all studies utilized mice with the C57BL/6 genetic background, the backcross to C57BL/6 was incomplete in the latter study (only 4 backcrosses) [25].

In summary, Nrf2 appears to protect the liver against steatosis by inhibiting lipogenesis and promoting fatty acid oxidation. This may be explained by the activation of ARE-containing transcription factors that regulate adipocyte differentiation and adipogenesis (e.g., CCAAT/enhancer-binding protein *β*, peroxisome proliferator-activated receptor-*γ*, aryl hydrocarbon receptor, and retinoid X receptor-*α*) and by the protection against redox-dependent inactivation of metabolic enzymes (e.g., 3-hydroxy-3-methylglutaryl-CoA reductase) [43], as well as by other mechanisms that remain unidentified. Future studies aimed at elucidating the molecular basis of these observations are warranted and authors need to take into account the interference of potential confounding factors. As reviewed herein, the role of Nrf2 on hepatic lipid processing in mice appears to be greatly dependent on the age of the animals, whereas factors such as mouse genetic background or gender do not appear to explain most of the contradictory findings in the literature (Table 1). It is tempting to speculate that the age-dependence of the Nrf2 regulation of hepatic lipogenesis may be a consequence of the progressive hepatic accumulation of lipids and/or the attenuation of the antioxidant defenses with age. This is supported by a work of Collins et al., who reported that old Ldlr^−/−^ mice (a model that mimics human NASH and atherosclerosis) suffer increased hepatocyte damage when fed a HFD. When compared with young animals, aged Ldlr^−/−^ mice on HFD showed a decline in the expression of antioxidant genes, which was directly related with a decrease in Nrf2 expression [65]. In addition, these animals displayed more severe hepatic steatosis, along with inflammation and fibrosis (NASH), while their younger counterparts simply developed fatty livers [66]. Studies of Nrf2 activation and/or deficiency in aged mice (>50 weeks of age) would be informative, especially since metabolic disorders are known to have a strong age-dependence.

## 7. Nrf2: A Therapeutic Target in NAFLD/NASH?

Despite its high prevalence in the industrialized world, there is currently no approved pharmacological treatment for NAFLD. It is crucial to develop and establish new options for the prevention and treatment of NAFLD/NASH. While there is still limited evidence in the literature that Nrf2 is activated in human subjects with NASH [67], Nrf2 deficiency has been repeatedly reported to favor the development of steatohepatitis and fibrosis in rodent models of NASH. In these studies, Nrf2-null animals developed many features of NASH when fed methionine- and choline-deficient (MCD) diet [18, 68, 69], high-fat diet [70], or high-fat and high-cholesterol diet [71]. These studies suggest that Nrf2 plays a key role in limiting the progression of NASH, which could be ascribed to the activation of antioxidative stress response genes but also to the modulation of fatty acid metabolism in hepatocytes. It would be important to assess the efficacy of Nrf2-activating compounds in preventing or treating NAFLD/NASH.

A great variety of thiol-reactive, electrophilic compounds isolated from dietary sources or plants are capable of activating Nrf2/ARE-dependent gene expression through inhibition of Keap1-mediated degradation [72–74]. Nrf2 inducing compounds can be grouped into different categories: (1) phenolic antioxidants (caffeic acid, epigallocatechin-3-gallate, and butylated hydroxyanisol); (2) dithiolethiones (oltipraz, 3H-1,2-dithiol-3-thione); (3) isothiocyanates (sulforaphane); and (4) triterpenoids (oleanolic acid). The synthetic triterpenoid 2-cyano-3,12-dioxooleana-1,9(11)-dien-28-oic acid (CDDO) and its derivative 1-[2-cyano-3-,12-dioxooleana-1,9(11)-dien-28-oyl]imidazole (CDDO-Im) are also potent inducers of Nrf2/ARE signaling [19, 75]. Studies demonstrating the* in vivo* chemopreventive and/or neuroprotective properties of oltipraz [74, 76], sulforaphane [77], and CDDO-Im [78] indicate a potential therapeutic usefulness for these Nrf2-activating molecules. In addition, new pharmacological Nrf2 activators have been synthesized in recent years, some of which have already entered the clinical trial stage [37, 79], including Protandim, dimethyl fumarate (BG-12), and CDDO-Me (bardoxolone methyl). Protandim (LifeVantage) is a dietary supplement that consists of five low-dose natural Nrf2 activators that activate Nrf2 through multiple kinase pathways [80]. BG-12/Tecfidera, an oral therapeutic agent containing dimethyl fumarate (Biogen Idec), has been recently approved for the treatment of multiple sclerosis [81]. Bardoxolone methyl (methyl 2-cyano-3,12-di-oxooleana-1,9(11)dien-28-oate) (Reata Pharmaceuticals) was shown to restore kidney function in patients with chronic kidney disease [82], although a recent phase III trial had to be terminated due to adverse effects (http://www.reatapharma.com/).

Whilst there have not been any clinical trials specifically on the effects of Nrf2 activation on liver disease, a number of studies have investigated the effects of Nrf2 activators in rodent models of NAFLD and NASH (Table 2). Oral administration of CDDO-Im, one of the most potent activators of Nrf2 in mouse liver known to date [83], prevented high-fat diet induced obesity and hepatic lipid accumulation in wild-type mice but not in Nrf2-null mice [19]. Likewise, oral CDDO-Me administration reduced hepatic lipid accumulation, lipogenic gene expression, and proinflammatory cytokine expression and ameliorated type 2 diabetes in mice fed HFD [26].

Sulforaphane [(−)-1-isothiocyanato-(4R)-methylsulfinylbutane], a natural-occurring isothiocyanate from cruciferous vegetables, strongly induces Nrf2 and ARE-mediated transcription activation through inhibiting Keap1-mediated Nrf2 degradation [84]. Treatment of rats with sulforaphane increases liver mitochondrial antioxidant defenses and protects from prooxidant-induced opening of the mitochondrial permeability transition pore [85]. A study by Oh et al. demonstrated that sulforaphane suppressed transforming growth factor-*β*-inducible expression of *α*-smooth muscle actin and profibrogenic genes in an immortalized human hepatic stellate cell line and attenuated bile duct ligation-induced liver fibrosis in mice [27]. In a study by Okada et al., long-term supplementation with sulforaphane suppressed the oxidative stress, inflammation, and hepatic fibrosis induced by MCD diet in mice [28]. Recently, Shimozono et al. [29] used two chemically distinct types of Nrf2 activator, namely, the dithiolethione oltipraz and a novel biaryl urea compound, termed NK-252 (1-(5-(furan-2-yl)-1,3,4-oxadiazol-2-yl)-3-(pyridin-2-ylmethyl)urea). The administration of both agents significantly reduced hepatic fibrosis in rats on a choline-deficient L-amino acid-defined diet. Whilst these studies suggest that Nrf2 activation presents new opportunities for treatment of NASH patients with hepatic fibrosis, it is important to bear in mind that NASH is closely related to overnutrition, insulin resistance, and obesity and not to a deficiency of amino acids such as methionine and choline [86]. Ideally, the use of Nrf2-activating compounds should be tested in animal models of NASH associated with obesity, insulin resistance, or dyslipidemia.

## 8. Conclusions

**Figure 2:**
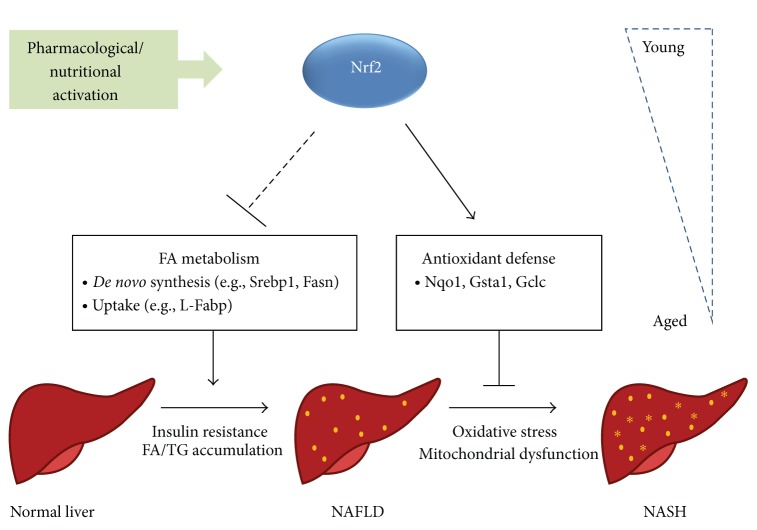
Schematic summary of the proposed protective roles of Nrf2 in nonalcoholic fatty liver disease (NAFLD). The progression from the simple accumulation of lipids in the hepatocyte to steatohepatitis (NASH) is depicted. NASH is associated with inflammation, fibrosis, and cirrhosis. The classical understanding is that Nrf2 coordinates the elimination of ROS and electrophiles derived from lipid peroxidation, thus preventing hepatocellular oxidative stress and mitochondrial dysfunction. In addition, there is growing evidence in the literature that Nrf2 regulates fatty acid metabolism by repressing genes that promote lipid accumulation in hepatocytes. In rodents, both mechanisms were shown to inhibit steatohepatitis in an age-dependent manner and can be induced via the pharmacological (e.g., CDDO-Im) or nutritional (e.g., sulforaphane) administration of Nrf2 activators.

A shematic overview of the proposed protective roles of Nrf2 in NAFLD is depicted in Figure 2. Rodent studies suggest that Nrf2 has a dual protective role in the progression from hepatic steatosis to steatohepatitis: (i) the negative regulation of genes that promote lipid accumulation in hepatocytes (“first hit”), likely by a combination of mechanisms that remain poorly understood; (ii) the activation of genes that promote the elimination of ROS and electrophiles derived from lipid peroxidation, thus preventing hepatocellular oxidative stress and mitochondrial dysfunction (“second hit”). Apparently, these protective mechanisms become less efficient with aging, which is expected to contribute to disease progression. The nutritional/pharmacological induction of Nrf2 signaling represents as a promising potential new strategy for the prevention and treatment of NAFLD/NASH.

## Figures and Tables

**Table 1:** The effect of Nrf2 activation/deficiency on hepatic lipid accumulation in C57BL/6 mice.

Gender	Age (weeks)	Hepatic lipid accumulation	Expression of FA synthesis genes	Reference
(a) Standard diet				
Male	8	Increased steatosis in Nrf2^−/−^. No difference in TGs	Higher in Nrf2^−/−^	[18]
Male	9	Reduced by Nrf2 activation	Reduced by Nrf2 activation	[19]
Male	10	Not reported	Higher in Nrf2^−/−^	[20]
Male	12	No difference in TGs	No difference	[21]
Male	19	Tendency for increased TGs in Nrf2^−/−^	Tendency for higher levels in Nrf2^−/−^	[22]
Male	25	No difference in TGs	No difference	[23]
(b) High-fat diet				
Male	12	Increased FFAs. No difference in TGs	Higher in Nrf2^−/−^	[21]
Male	19	Tendency for increased TGs in Nrf2^−/−^	No difference	[22]
Female	21	Reduced lipid content by Nrf2 activation	Reduced by Nrf2 activation	[24]
Male	25	No difference in TGs	No difference	[23]
Male	26	Reduced total lipid and TGs in Nrf2^−/−^	Lower in Nrf2^−/−^	[25]

FFA, free fatty acids; TG, triglyceride.

**Table 2:** The effects of Nrf2 activators in rodent models of NAFLD or NASH.

Nrf2 activator	Species	Administration route	Reported effect	Reference
CDDO-Im	Mouse	Oral	Prevented HFD-induced increases in body weight, adipose mass, and hepatic lipid accumulation	[24]
CDDO-Me	Mouse	Oral	Reduced hepatic lipid accumulation, proinflammatory cytokine expression, and lipogenic gene expression in mice fed HFD	[26]
Sulforaphane	Mouse	Intraperitoneal	Attenuated hepatic fibrosis induced by bile duct ligation	[27]
Sulforaphane	Mouse	Oral	Suppressed oxidative stress and hepatic fibrosis induced by MCD diet	[28]
Oltipraz, NK-252	Rat	Oral	Attenuated hepatic fibrosis induced by CDAA diet	[29]

CDAA, choline-deficient L-amino acid-defined diet; CDDO-Im, 1-[2-cyano-3-,12-dioxooleana-1,9(11)-dien-28-oyl]imidazole; CDDO-Me, 1-[2-cyano-3-,12-dioxooleana-1,9(11)-dien-28-oyl]methyl; HFD, high-fat diet; MCD, methionine- and choline-deficient diet.

## Abbreviations

ARE: Antioxidant response element
CDDO-Im: 1-[2-Cyano-3-,12-dioxooleana-1,9(11)-dien-28-oyl]imidazole
CDDO-Me: 1-[2-Cyano-3-,12-dioxooleana-1,9(11)-dien-28-oyl]methyl
ER: Endoplasmatic reticulum
FA: Fatty acid
FFA: Free fatty acid
GSH: Glutathione
HFD: High-fat diet
MCD: Methionine- and choline-deficient
NAFLD: Nonalcoholic fatty liver disease
NASH: Nonalcoholic steatohepatitis
ROS: Reactive oxygen species
TG: Triglyceride
UPR: Unfolded protein response
VLDL: Very low density lipoprotein.
